# Determinants of exclusive breastfeeding practices among mothers in azezo district, northwest Ethiopia

**DOI:** 10.1186/s13006-016-0081-x

**Published:** 2016-08-02

**Authors:** Mulusew Andualem Asemahagn

**Affiliations:** School of Public Health, College of Medicine and Health Sciences, Bahir Dar University, Bahir Dar town, Ethiopia

**Keywords:** Infants, Exclusive breastfeeding, Factors, Azezo District, Ethiopia

## Abstract

**Background:**

Exclusive breastfeeding (EBF) is a very important, long lasting and cost effective intervention to help reduce the morbidity and mortality of infants. However, a large proportion of infants are not exclusively breastfed as recommended by the World Health Organization. The study aim was to assess the exclusive breastfeeding practices and identify determinants among mothers in Azezo District, Ethiopia.

**Methods:**

A community based cross-sectional study was conducted among 346 mothers with infants aged between 0–6 months. Data were collected using a pretested, interviewer administered questionnaire. Descriptive statistics and logistic regression analysis were used to describe the study objectives and identify the determinants of EBF in the previous 24 h. Associations between the study and outcome variables were described using odds ratios and 95 % confidence intervals (CI).

**Results:**

Any breastfeeding and exclusive breastfeeding in the previous 24 h were 99 and 79 %, respectively. The mean number of exclusive breastfeeds in the 24 h period was 6.5. Exclusive breastfeeding rates were highest among mothers aged ≥ 30 years (Adjusted odds ratio [AOR] 1.75; 95 % CI 1.14, 3.42). Infants whose mothers were unemployed were more likely to be exclusively breastfed than infants whose mothers were employed (AOR 1.62; 95 % CI 1.03, 2.95). Mothers earning ≤ 1000 birr (US $47.62) monthly were 77 % less likely to practice EBF (AOR 0.23; 95 % CI 0.13, 0.44). Mothers who delivered at the healthcare facility practised more exclusive breastfeeding than those who delivered at home (AOR 2.18; 95 % CI 1.22, 4.35). Mothers who received antenatal and postnatal care had better rates of EBF (AOR 2.24; 95 % CI 1.18, 5.76 and AOR 1.62; 95 % CI 1.09, 3.21) and mothers not practicing prelacteal feeding were more likely to exclusively breastfeed compared with mothers practicing prelacteal feeding (AOR 2.16; 95 % CI 1.16, 4.06).

**Conclusions:**

Any breastfeeding and exclusive breastfeeding rates in the previous 24 h are relatively high in the study area compared with previous studies. Maternal factors (age, education, income, employment, antenatal care, prelacteal feeding), infants’ age, delivery place and information access were independent predictors to EBF in previous 24 h. Improving the mother’s knowledge, income, information access, nutritional counselling, quality of antenatal and postnatal care service, place of delivery and avoiding prelacteal feeding practices are important to improving the exclusive breastfeeding rate in the previous 24 h.

## Background

The World Health Organization (WHO) recommends the practice of exclusive breastfeeding (EBF) for the first six months in addition to its continuation with the addition of supplementary foods, for 2 years or more [1]. Exclusive breastfeeding is the practice of feeding on infants only breast milk for the first six months [1, 2]. It is the most cost effective intervention to reduce infant morbidity and mortality worldwide [2–4]. Exclusive breastfeeding is crucial since human milk contains nutrients, living cells and defensive factors which enable infants to have a better immunity, physical and mental development [5, 6].

However, more than 85 % of mothers worldwide did not follow WHO recommendation and only 35 % of infants younger than four months was exclusively breastfed [7]. Evidence shows that the majority of mothers started to EBF their infants at birth and the rate declined greatly about two or more months [1, 4, 6, 7]. Almost half of the 10 million deaths of children younger than 5 years old every year are direct or indirect consequences of malnutrition. Many of those deaths are associated with inappropriate breastfeeding practices [7, 8].

A cross-sectional study conducted in 2010 from Southern Brazil [9] showed a 72.5 % overall breastfeeding rate, and 43.7 and 53.9 % EBF among infants younger than six and four months of age, respectively. Based on another study from Al-Hassa, Saudi Arabia [10], only 24.4 % infants were exclusively breastfed at the age of six months. In India, the 462 mothers in this study did EBF their infants at birth, and the rate was 97.0 % at 3 months and 62.0 % up to six months of age [11]. Similarly, evidence from Canada indicated an overall exclusive breastfeeding rate of 90.3 % at birth and 13.8 % up to six months of age [12].

Despite several interventions, the rate of EBF in the previous 24 h continues to be inadequate in developing countries [8, 13–17]. Based on a study from rural Africa [14], EBF prevalence was varied among HIV and non HIV mothers. Exclusive breastfeeding rates at three and five months were 83.1 and 76.5 %, respectively among HIV negative women and 72.5 and 66.7 %, respectively among HIV positive women.

Evidence from Ghana [14] revealed that 99.7 and 51.6 % of mothers practiced EBF and non-exclusive breastfeeding. Other studies show that less than half (19 and 48 %) of women exclusively breastfed their infants up to the ages of four months in Tanzania and Uganda, respectively [15]. Another study from Uganda [16] also revealed that 99.0 % mothers practiced breastfeeding at birth and the rate of EBF was 7 and 0 %, at three and six months, respectively. In Western Tanzania the rate of EBF was 58.0 % in previous 24 h [17]. About 96.0 and 18.0 % of mothers EBF at birth and practised EBF up to six months, respectively, in South Africa [18]. In Sudan [19], 64.5 and 9.5 % of mothers practiced EBF at four and for six months, respectively. In Egypt, 95.8 % mothers exclusively breastfed at birth and 9.7 % EBF for 6 months [20].

In Ethiopia, there is inadequate evidence on exclusive breastfeeding rates in previous 24 h. Considering the high percentage of improper infant feeding practices in Ethiopia, the value of EBF and the objective of Millennium Development Goals (MDGs), the Ethiopian Government adopted the Infant and Young Child Feeding (IYCF) guideline in 2004 [21]. Even though various interventions were introduced at the health facility and at the community levels, there has been no significant change observed regarding EBF in the previous 24 h. This might be due to disorganized efforts, poor data management, and an inability to measure the performance and identify hindering factors through research.

In 2009, in Ethiopia, the EBF rates were 56.7 and 49.0 % for the first four and six months respectively [22]. The 2011 Ethiopian Demographic and Health Survey (EDHS) [23] showed a rate of EBF of 52.0 % during the first six months after birth. Evidence from Southeast Ethiopia [24] showed a 71.3 % EBF rate. As far as we know, EBF in the previous 24 h and associated factors (the aim of this study) have not been studied in Azezo District, Northwest Ethiopia. In addition, the presence of more than five high and medium level factories within the district with a high local employment sourced, it is believed to have an influence on the rate of EBF in the previous 24 h. Hence, the aim of this study is to provide important evidence on the EBF status and determinant factors to local administrators, North Gondar Zone Health Department, Amhara Regional Health Bureau and the other researchers interested in breastfeeding.

## Methods

A community based cross-sectional study was conducted in Azezo District, Northwest Ethiopia, from May–June 2014. Azezo District is located 12 km south of Gondar and about 718 km to the northwest of Addis Ababa, the capital city of Ethiopia. It has seven Kebeles (local administrations). Based on the EDHS 2005, the District has an estimated population of 54, 434 of which 45 % are males [25]. Azezo District health facilities comprise one health center, three private clinics and five pharmacy centers. Azezo is a trade commercial center, is directly connected to Metema, and has more than six medium level factories, like the Guna shoe factory, Dashen brewery factory and others.

All women having children aged from 0–6 months in Azezo District during the study period were sourced to this study. The sample size was calculated using Epi Info Version20 based on the following assumptions: EBF prevalence (*p*) 71.3 % [24]; 95 % CI ± 1.96, precision error (d) 4 % and allowing for a 10 % non-response contingency. We aimed for a final sample size of 540. Unfortunately, the actual number of mothers with infants 0–6 months in the district was only 346, based on the evidence from Health Extension Workers and so we decided to include all mothers having 0–6 month old infants in our study. To contact each mother, we took all the necessary information such as Kebeles (local administrations), house numbers and phone numbers from the Health Extension Workers office.

An structured interviewer administered questionnaire was used to collect data from the study participants. The questionnaire was developed by referring to other studies showing exclusive breastfeeding rates and included the WHO exclusive breastfeeding definition [1, 21–24, 26]. The data collection tool was first prepared in English and translated to Amharic (local language) and back to English to check its consistency. Data on socio-demographic factors, breastfeeding practices, birth intervals, parity, antenatal care visits (ANC), postnatal care (PNC) services, access to information about EBF and place of birth were major components of the questionnaire. It was pretested among mothers having 0–6 month infants in Gondar Town to assure its validity. Gondar town is 12 km from Azezo District, with similar healthcare setups and a similar population.

Seven, second year nursing students (one per Kebele) were recruited as data collectors. The author provided a 1 day training to the data collectors on the study objectives, data collection procedures and data confidentiality issues.

Ethical clearance was obtained from the Ethical Review Committee of Amhara Regional Health Bureau research and technology transfer core process owner. A supporting letter was obtained from Azezo District administration office. Verbal consent was given by each woman in the study, after explaining study objectives, data collection procedures and data confidentiality issues. Participation was fully voluntary based including the right to withdraw from participation if they feel uncomfortable during the process. To insure data confidentiality, no one other than the investigators had access to the data and data were used only for describing the stated objectives.

Data collectors collected data through face to face interviewing of mothers with infants aged from 0–6 months old. The principal investigators provided supportive supervision to the data collectors. Data were checked daily for completeness and consistency. Data were edited and analyzed using Statistical Package Software for Social Science (SPSS) Version20. Descriptive statistics were used to describe the study participants and the practice of EBF in previous 24 h. Exclusive breastfeeding was calculated by accepting infants below 6 months who were fed only breast milk in the 24 h preceding the survey as numerator, and the total number of infants within the same age group (0–6 months) as denominator. Binary logistic regression analysis was made to each independent variable to EBF in previous 24 h. Finally, multivariable logistic regression was computed on independent variables tested by binary regression analysis to determine independent predictors of outcome variable. Odds ratio at CI and *p*-value < 0.05 were used to describe significance and strength of association between the study and outcome variables.

## Results

### Sociodemographic characteristics of mother-child pairs

From the total 346 mothers with infants aged 0 to 6 months, 332 responded to the questionnaire (response rate of 96 %). More than half (63.0 %) of mothers were aged less than 30 years. The majority of mothers, 242 (73.0 %) were Orthodox Christian followers. Most of the mothers (87.0 %) were Amhara in ethnicity. Nearly half (48.2 %) the mothers did not attend formal education (from grade 1 to higher education). Most mothers were married (72.0 %) and 67.0 % were unemployed. Less than half (41.0 %) of them earned ≤ 1000 Ethiopian birr (US $47.62) monthly (Table 1).

**Table 1:** Sociodemographic variables of mothers and infants in Azezo District, Northwest Ethiopia, 2014

Variables	Category	Frequency	Percent (%)
Age in year	≤30 >30	180 152	63.0 37.0
Religion	Orthodox Muslim	242 90	73.0 27.0
Ethnicity	Amhara Tigre Oromo	289 33 10	87.0 10.0 3.0
Education status	Informal education^a^ Formal education	160 172	48.2 51.8
Marital status	Single Married	92 240	28.0 72.0
Occupation	Housewife Merchant Governmental employee Non-governmental employee Daily labourer	111 90 83 26 22	33.4 27.0 25.0 8.0 6.6
Sex of infant	Male Female	133 199	40.0 60.0
Age of infant	≤3 months 3–6 months	151 181	45.5 54.5
Monthly income	≤1000 birr >1000 birr	136 196	41.0 59.0

^a^Informal education: type of education where there is no regular class, it is a form of mass education to enable adults to read and write

### Obstetric care, prenatal and postnatal care and access to information

More than half (56 %) of studied mothers had 1–2 birth intervals respectively. A large number, 300 (90.0 %) of mothers attended antenatal visits and three-quarters received advice on EBF there. Nearly half (49.0 %) of the mothers received postnatal care and counselling. Only 70 (15.0 %) of the interviewed mothers practiced prelacteal feeding, the reasons given were; being busy, less knowledge on EBF and an assumption that prelacteal feeding is more important to infants than EBF.

The number of exclusive breastfeeds in the previous 24 h was 6.5. Over one third of mothers (36.0 %) reported accessing the available information on EBF and their major information sources were health facilities (78.8 %). Almost three-quarters (76.0 %) of mothers delivered vaginally. The rates of EBF were 79 % and mixed breastfeeding in Azezo District was 99 % (Table 2). Exclusive breastfeeding was inversely proportional with the infants’ age. Exclusive breastfeeding rates were 79, 62.0, 52, 43.0 and 29 % at ≤ second, third, fourth, fifth and 6th months of infants’ age, respectively.

**Table 2:** Obstetric care, antenatal/postnatal care and information on EBF (exclusive breastfeeding) practices in Azezo District, Northwest Ethiopia, 2014

Variables	Category	Frequency	Percent (%)
Parity of mothers	1–2 >3+	142 190	75.0 25.0
Birth interval (year)	1–2 3–4	185 147	56.0 44.0
Antenatal care received	Yes No	300 32	90.0 10.0
Number of antenatal visits	1 2–3 =4	90 123 87	30.0 41.0 29.0
Received information on EBF during antenatal care	Yes No	228 72	76.0 24.0
Postnatal counselling	Yes No	162 170	49.0 51.0
Prelacteal feeding:	Yes No	70 262	15.0 79.0
Information availability on EBF	Yes No	120 212	36.0 64.0
Information source on EBF	Health facility Non health facility	258 74	78.8 22.2
Place of delivery	Home Public facility Private facility	70 195 67	21.0 59.0 20.0
Type of delivery	Normal/vaginal Caesarean	251 81	76.0 24.0
Initiated breastfeeding in first hour	Yes No	262 66	79.0 21.0
Breastfeeding practices	Yes No	329 3	99.0 1.0

### Factors affecting exclusive breastfeeding practices

The bivariable and multivariable logistic regression analysis revealed that maternal related (age, education status, employment status, monthly income, place of delivery, antenatal visit), infants’ age, information on EBF, prelacteal feeding practices and availability of resources were all independent predictors to EBF practices in previous 24 h (Table 3).

**Table 3:** Factors affecting exclusive breastfeeding among mothers in Azezo District, Northwest Ethiopia, 2014 (*n* = 332)

Variables	Exclusive breastfeeding		Crude Odds Ratio (95 % CI)	Adjusted Odds Ratio (95 % CI)
	Yes (%)	No (%)		
Mothers’ age (years)				
<30 ≥30	110 (33.0) 152 (46.0)	42 (12.6) 28 (8.4)	1 2.07 (1.17, 3.68)	1 1.75 (1. 14, 3.42)
Infants’ age (months)				
≤3 3–6	129 (39.0) 133 (40.0)	22 (6.6) 48 (14.4)	2.12 (1.17, 3.85) 1	1.86 (1.15, 3.26) 1
Religion				
Orthodox Muslim	194 (58.4) 69 (21.0)	48 (14.6) 21 (6.0)	1.23 (0.66, 2.28) 1	0.78 (0.45, 1.79) 1
Educational status				
Informal education Formal education	118 (35.5) 144 (43.4)	42 (12.7) 28 (8.4)	0.55 (0.31, 0.97) 1	0.49 (0.28, 0.89) 1
Marital status				
Single Married	60 (18.0) 202 (61.0)	32 (9.6) 38 (11.4)	0.35 (0.20, 0.63) 1	0.32 (0.39, 1.52) 1
Employment status				
Employed Unemployed	78 (23.5) 184 (55.4)	31(9.1) 39 (12.0)	1 1.88 (1.05, 3.33)	1 1.62 (1.03, 2.95)
Infants’ sex				
Male Female	95 (28.6) 167 (50.3)	38 (11.5) 32 (9.6)	0.48 (0.27, 0.84) 1	0.52 (0.32, 1.73) 1
Monthly income(birr)				
≤1000 >1000	90 (27.0) 172 (52.0)	46 (14.0) 24 (7.0)	0.25 (0.15, 0.49) 1	0.23 [0.13, 0.44] 1
Parity of mothers				
1–2 3 and above	115 (35.0) 147 (44.0)	27 (8.0) 43 (13.0)	1.25 (0.7, 2.21) 1	1.18 (0.56, 1.99) 1
Birth interval (year)				
1–2 3–4	140 (42.0) 122 (37.0)	45 (13.5) 25 (7.5)	0.64 (0.36, 1.14) 1	0.58 (0.31, 1.12) 1
Type of delivery				
Vaginal Caesarean	203 (61.0) 59 (18.0)	48 (14.4) 22 (6.6)	1.58 [0.85, 2.93] 1	1.26 (0.78, 2.65) 1
Place of birth				
Home Healthcare facility	46 (14.0) 216 (65.0)	24 (7.0) 46 (14.0)	1 2.45 (1.31, 4.59)	1 2.18 (1.22, 4.35)
Antenatal care				
Yes No	243 (73.0) 19 (6.0)	57 (17.0) 13 (4.0)	2.92 (1.27, 6.64) 1	2.24 (1.18, 5.76) 1
Received information on EBF during pregnancy				
Yes No	206 (69.0) 56 (19.0)	22 (7.0) 16 (5.0)	2.68 (1.24, 5.74) 1	1.98 (1.18, 5.20) 1
Postnatal care:				
Yes No	137 (41.0) 125 (38.0)	25 (7.5) 45 (13.5)	1.97 (1.11, 3.53) 1	1.62 (1.09, 3.21) 1
Prelacteal feeding				
Yes No	47 (14.0) 215 (65.0)	23 (7.0) 47 (14.0)	1 2.24 (1.19, 4.20)	1 1.92 (1.12, 3.85)
Access to information				
Yes No	212 (64.0) 50 (15.0)	46 (14.0) 24 (7.0)	2.21 (1.19, 4.12) 1	2.16 (1.16, 4.06) 1

Mothers whose age was ≥ 30 years were more likely to exclusively breastfed infants compared with mothers < 30 years old (OR 2.07; 95 % CI 1.17, 3.68). Infants ≤ 3 months old were more likely to be exclusively breastfed than infants within 3–6 months age range (OR 2.12; 95 % CI 1.17, 3.85). Mothers with informal education and earning a monthly income of ≤ 1000 Ethiopia birr (US $47.62) were 54.0 and 77.0 % less likely to practice EBF than their counter parts, respectively. Unemployed mothers were more likely to practice better EBF than employed mothers (OR 1.88; 95 % CI 1.05, 3.33). Mothers who delivered at healthcare facility and those who had antenatal care exclusively breastfed their infants more than mothers who delivered at home and those who did not have antenatal care (OR 2.45; 95 % CI 1.31, 4.59 and OR 2.92; 95 % CI 1.27, 6.64), respectively. Infants from mothers who did not practice prelacteal feeding were more likely to be EBF (OR 2.24; 95 % CI 1.19, 4.20) (Table 3).

## Discussion

Exclusive breastfeeding is very important and an economical way of feeding babies worldwide. This study revealed a 99.0 % initiation rate of breastfeeding which is similar to studies conducted in India [11], Ghana [14], Egypt [20], Uganda [16], South Africa [18] and Ethiopia [22, 24] where other breastfeeding practices were 100, 99.7, 95.8, 99.0, 96.0 and 96.0–98.0 % respectively. Possible reasons for this high rate could be community acceptance and involvement, government concern, health facilities participation and the increased efforts to achieve the 2015 Millennium Development Goals (MDGs), in relation to child and maternal health since it is the world’s primary agenda. However, this finding is higher than the study findings from Brazil [9] where non-exclusive breastfeeding in previous 24 h was 72.5 %. The probable reasons to this discrepancy could be a variation in study period and the increased effort made in Ethiopia to achieve MDGs as Ethiopia achieved the maternal and child health component of MDGs before 2015.

This study also revealed a 79 % EBF rate in previous 24 h, which is in line with study findings from Jordan [27], South Africa [14], Ghana [7], Ethiopia [24, 28], where EBF in previous 24 h were 70, 76.5, 79, 71.3 and 81 %, respectively. On the other hand, this finding of 79 % in Azezo District was less when compared with study findings from India [11], where the EBF practice among infants at 3 months was 97 and 62 % at 6 months. The reasons for this variation between Ethiopia and India could be differences in the level of community awareness, access to information on EBF, maternal education, quality of antenatal care, health professionals’ commitment, access to health facility, culture, monthly income and provision of postnatal care .

The rate of EBF in previous 24 h is higher when compared to study findings from the Al Hassa-Soudi Arabia [10]: 24.4 %, Brazil [4, 9]:39 and 43.7 %, Uganda [16]:7 %, Egypt [20]: 9.7 %, Tanzania [17]: 58 %, Ghana [14]: 51.6 % and South Africa [18]:18 %. Possible justifications for this variation could be the study period and increased Ethiopian Government efforts to improve maternal and child health through a community based Health Extension Program. It is also due to community involvement, increased number of health facilities/health professionals every year, advice given at antenatal and postnatal care clinics (90 % mothers visited the antenatal clinic) and the high use of delivering at a healthcare facility (79 %) (Table 2).

It is also higher compared with previous studies conducted in Ethiopia: 56.7 and 49 % at four and 6 months in Oromia Region [22] and 52 % national EDHS 2011 survey [23]. Logical reasons to this variation are differences in study period and community awareness (76 % received advice at antenatal clinics, 90 % visited antenatal clinics and 79 % delivered at the healthcare facility).

The logistic regression analysis showed that older mothers (≥30 years) practiced more EBF (OR 2.07; 95 % CI 1.17, 3.68) than mothers < 30 years of age. The reason could be that as the maternal age increased, infant management experiences will also be increased. Younger mothers also thought their breast size and beauty will be affected if they practiced EBF for a longer time, hence they usually give the infant rearing responsibilities to servants and started supplementary feeding early.

Younger infants ≤ 3 months age were (OR 2.12; 95 % CI 1.17, 3.85) more to be exclusively breastfed than infants aged between 4–6 months. Several studies [9, 12, 14, 16–18, 22–24, 28] also strengthen this correlation, where infants’ EBF and age are inversely related. The logical explanation could be the commitment of mothers to either home or office works and the introduction of supplementary feedings. Mothers with no and informal education were 45 % less likely to exclusively breastfeed their infants compared with their counter parts (Table 3). Research findings [4, 7, 9, 12, 22, 23, 29] supported this, where lower maternal education was predictor to lower EBF in previous 24 h. It is true that non educated mothers do not have the scientific knowledge and have difficulty understanding the written messages and antenatal care advice on EBF. Hence, they will introduce supplementary feeding to their infants early by assuming it is good compared with the formally educated mothers.

In the case of employment status, unemployed mothers practiced relatively better EBF than their counter parts (OR 1.88; 95 % CI 1.05, 3.33), which is in agreement with study findings from Saudi Arabia [10], Brazil [4] and Ethiopia [22–24, 28]. Employed mothers may be relatively overloaded with their office and home activities so may have limited contact time with infants. Low family income can hinder EBF practices in the previous 24 h; 75 % lower than EBF in mothers with higher income (Table 3). It is also supported by evidences from Brazil [4] and Ethiopia [22, 28].

Justification to this could be that mothers with a low family income spend most of their time working to gain additional money to feed their families, and so may start early supplementary feeding.

The exclusive breastfeeding practice of mothers who delivered at healthcare facilities was double compared with mothers who delivered at home (OR 2.45; 95 % CI 1.31, 4.59). It is supported with study findings from Tanzania [17], Ghana [14] and Ethiopia [28, 29]. Logical reasons could be the impact of information on EBF that they received from the healthcare facilities. However, this finding differed with findings from Canada [12] where mothers delivered at home were more likely to remain exclusively breastfeeding for 6 months.

Frequent antenatal and postnatal care visits showed significant association with EBF in previous 24 h (Table 3), which was also true at different studies [13, 20, 29, 30]. Possible justification could be the increased knowledge and attitudinal changes due to the information provided by the antenatal care clinics on infant feeding and the nutritional values of breast milk. Similarly, mothers who did not practice prelacteal feeding were twice as likely to EBF in previous 24 h than the respective groups. Prelacteal feeding will prevent the infants to take an adequate amount of breast milk with the appropriate frequency. This will result in poor EBF in previous 24 h and different outcomes in infants’ health status. Infants who are never received any supplements are most likely to be exclusively fed on maternal milk [3, 10, 16, 24].

## Conclusions

The incidence of exclusive breastfeeding and other breastfeeding practices of mothers with 0–6 month old infants in Azezo District are high compared with previous study findings. The number of exclusive breastfeeds in 24 h is below the WHO infant and young child feeding recommendation. Maternal factors (age, education, income, employment, antenatal care service, no prelacteal feeding), infants’ age, place of birth and access to information were determinants to EBF practices in previous 24 h. Improving women knowledge, income, access to information, nutritional counselling, quality of antenatal and postnatal care, place of birth and avoidance of prelacteal feeding are important to improve EBF in previous 24 h.

## Abbreviations

AOR, adjusted odds ratio; ANC, antenatal care: CI, confidence interval; COR, crude odds ratio; EDHS, Ethiopian Demographic and Health Survey; EBF, exclusive breastfeeding; HIV, Human Immune Deficiency Virus; IYCF, Infant and Young Child Feeding; MDGs, Millennium Development Goals; PNC, postnatal care; SPSS, Statistical Packages for Social Sciences; WHO, World Health Organization
